# Elevated triglycerides rather than other lipid parameters are associated with increased urinary albumin to creatinine ratio in the general population of China: a report from the REACTION study

**DOI:** 10.1186/s12933-019-0863-8

**Published:** 2019-05-04

**Authors:** Yu-Xia Wang, An-Ping Wang, Ying-Nan Ye, Zheng-Nan Gao, Xu-Lei Tang, Li Yan, Qin Wan, Wei-Qing Wang, Zuo-Jie Luo, Gui-Jun Qin, Lu-Lu Chen, Yi-Ming Mu

**Affiliations:** 1 School of Medicine, Nankai University, No. 94 Weijin Road, Tianjin, 300071 China; 2 Department of Endocrinology, Chinese People’s Liberation Army General Hospital, No. 28 Fuxing Road, Beijing, 100853 China; 3 Department of Endocrinology, Dalian Municipal Central Hospital, No. 826 Southwest Shahekou District Road, Dalian, 116033 China; 4 Department of Endocrinology, First Hospital of Lanzhou University, No. 1 Donggang West Road, Lanzhou, 730000 China; 5 Department of Endocrinology, Sun Yat-sen Memorial Hospital, Sun Yat-sen University, No. 107 Yanjiang West Road, Guangzhou, 510120 China; 6 Department of Endocrinology, Affiliated Hospital of Luzhou Medical College, No. 25 Taiping Road, Luzhou, 646000 China; 7 Department of Endocrinology, Ruijin Hospital, Shanghai Jiaotong University School of Medicine, No. 197 Ruijin 2nd Road, Shanghai, 200025 China; 8 Department of Endocrinology, First Affiliated Hospital of Guangxi Medical University, No. 6 Shuangyong Road, Nanning, 530021 China; 9 Department of Endocrinology, First Affiliated Hospital of Zhengzhou University, No. 1 Jianshe Road, Zhengzhou, 450052 China; 10 Department of Endocrinology, Union Hospital, Tongji Medical College, Huazhong University of Science and Technology, No. 1277 Jiefang Avenue, Wuhan, 430030 China

**Keywords:** Lipid parameters, Urinary albumin to creatinine ratio, Triglycerides, General population

## Abstract

**Background:**

Dyslipidaemia has always been regarded as the cornerstone of arteriosclerosis and is related to the pathogenesis of renal insufficiency. However, it is unclear which routinely available lipid parameter is related to urinary albumin to creatinine ratio (UACR). The purpose of this study was to examine the lipid abnormalities associated with UACR in the general population in China.

**Methods:**

The present study was nested in an ongoing Risk Evaluation of cAncers in Chinese diabetic Individuals: A lONgitudinal (REACTION) study, which was designed to demonstrate the association of abnormal glucose metabolism with the risk of cancer in the Chinese population. This cross-sectional study included 34, 569 subjects (11, 390 males and 23, 179 females) from 8 different regional community cohorts, with an average age of 57.9 years. The UACR data were divided into the < 25% group, the 25–49% group, the 50–74% group, and the ≥ 75% group according to the quartile division of the centre where the subjects visited. The lipid classes were defined according to the guidelines for the prevention and treatment of dyslipidaemia in Chinese adults. Multiple logistic regression analysis was used to evaluate the association of the lipid parameters and UACR.

**Results:**

Multivariable regression analysis revealed that compared with the other lipid parameters, triglycerides (TG) showed an adjusted odds ratio that was significant in model 1–4. This relationship was attenuated after adjusting for Haemoglobin A1c (HbA1c) and blood pressure (BP), but TG ≥ 2.3 mmol/L was still significantly associated with UACR in total subjects and in both men and women (OR: 1.131, 95% CI 1.065–1.203, P < 0.001 in total subjects; OR: 1.134, 95% CI 1.022–1.258, P = 0.017 in men; OR: 1.129, 95% CI 1.046–1.219, P = 0.002 in women). In the stratified analysis, elevated TG was significantly associated with increased urinary albumin in subjects with eGFR ≥ 90 mL/min per 1.73 m^2^, 5.6 ≤ FBG < 7.0 or 7.8 ≤ PBG < 11.1 mmol/L, 24 ≤ BMI < 28 kg/m^2^, 120 ≤ SBP < 140 and/or 80 ≤ DBP < 90 mmHg.

**Conclusions:**

We conclude that high TG levels rather than total cholesterol, low-density lipoprotein cholesterol, high-density lipoprotein cholesterol, or non-high-density lipoprotein cholesterol levels are associated with UACR in the general population in China.

**Electronic supplementary material:**

The online version of this article (10.1186/s12933-019-0863-8) contains supplementary material, which is available to authorized users.

## Background

An elevated level of the urinary albumin to creatinine ratio (UACR) is not only a marker of renal dysfunction but also has been described as an independent predictor of cardiovascular disease in diabetic [1, 2] and non-diabetic patients [3, 4]. Moreover, recent findings have indicated that the UACR is more closely associated with diabetic retinopathy than the estimated glomerular filtration rate (eGFR) [5] and might be valuable in evaluating the risk for cognitive decline [6, 7]. Additionally, a robust body of literature has demonstrated that moderately increased albuminuria (UACR less than 30 mg/g) within the accepted normal range is associated with higher cardiovascular morbidity and mortality even in the general population [8, 9]. Moreover, every 3.01 mg/g (equivalent to 0.4 mg/mmol) increment in UACR conferred a 5.9% increase of major cardiovascular events [10].

The mechanisms by which increased UACR is linked to an increased risk of cardiovascular disease remain to be clarified, but one of the mechanisms is its link with atherogenic lipoproteins. Dyslipidaemia has always been regarded as the cornerstone of arteriosclerosis and the primary target of therapy according to international guidelines [11, 12]. In addition, it has been reported that dyslipidaemia associated with arteriosclerotic complications is the most common cause of death in chronic kidney disease (CKD) patients, and dyslipidaemia has also been shown to be an independent risk factor for the progression of CKD [13, 14]. Furthermore, a previous meta-analysis demonstrated that statins for treatment of dyslipidaemia may be beneficial for the reduction of albuminuria in CKD patients [15]. In practice, however, it is unclear which routinely available lipid measure is more applicable in estimation of kidney function. Research on this aspect has yielded controversial results, and most of these studies were in people with diabetes [16, 17]. Recently, a cross-sectional study was conducted in China to investigate the association between lipid parameters, albuminuria and chronic kidney disease. However, the study included only 9730 subjects, and the authors did not adjust for serum creatinine (Cr) and liver function in the logistic regression, which were thought to potentially skew results [18].

Lipids remain part of the conventional risk factors for UACR and atherosclerosis, especially in the general population. Therefore, the aim of this study was to comprehensively assess the association of all routine lipid parameters and albuminuria in the general population in 8 different regions of China.

## Methods

### Study population and design

**Fig. 1:**
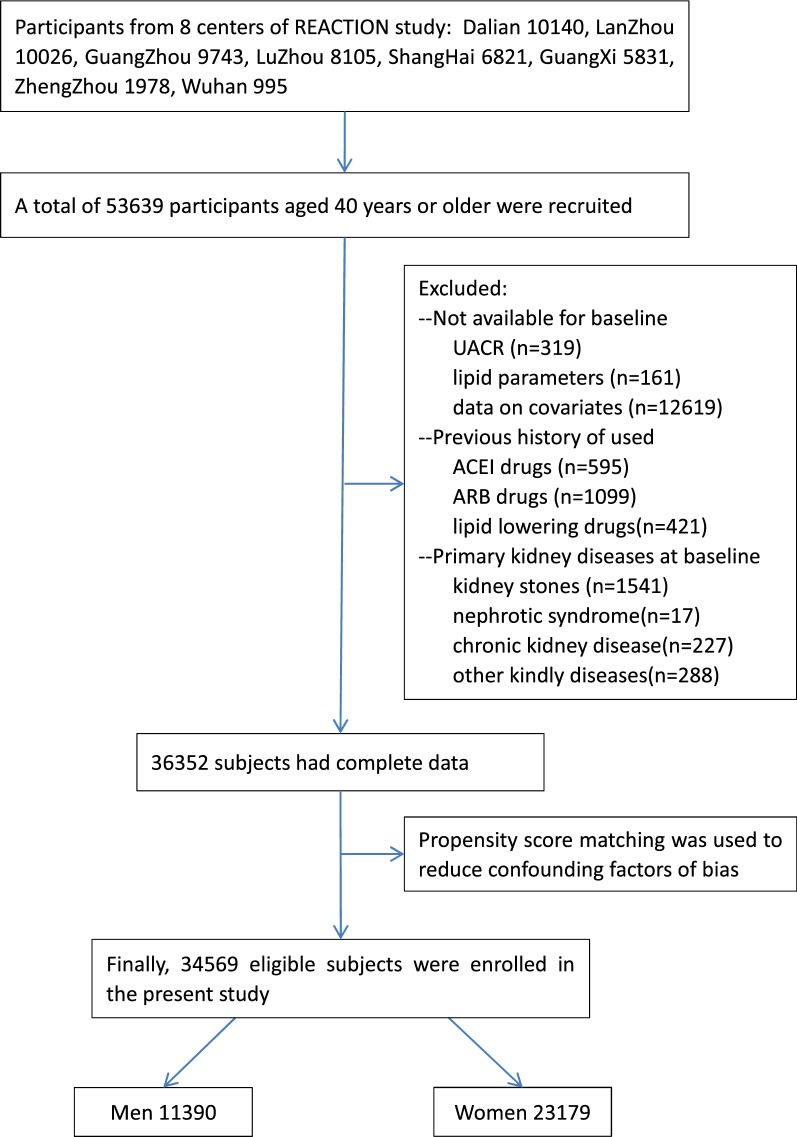
Flow chart of the selection of study participants

The present study was nested in A lONgitudinal REACTION (Risk Evaluation of cAncers in Chinese diabeTic Individuals) study, which was designed to investigate the association of type 2 diabetes mellitus (T2DM) and pre-diabetes with the risk of cancer in the Chinese population, described previously [19]. The REACTION study was set up as a multicentre prospective observational study, and our study population was from the eight of the centres. A total of 53, 639 participants aged 40 years or older were recruited and invited to participate by questionnaire survey between March and December 2012. (Dalian 10140, Lanzhou 10026, Guangzhou 9743, Luzhou 8105, Shanghai 6821, Guangxi 5831, Zhengzhou 1978, Wuhan 995). Subjects who had been diagnosed with primary kidney diseases, used ACEI/ARB medicines, used lipid-lowering drugs or whose data missing and/or included outliers were excluded. A total of 36, 352 subjects had complete data. After the propensity score matching, the remaining 34, 569 eligible subjects (11, 390 males and 23, 179 females) were enrolled in this cross-sectional study (Fig. 1).

All investigators received extensive training related to the study questionnaire and outcome measures before carrying out the investigation. The study protocol was approved by the Committee on Human Research at Rui-Jin Hospital affiliated with the School of Medicine, Shanghai Jiao Tong University. Written informed consents were obtained from all participants before data collection.

### Clinical evaluation and laboratory measurements

All participants received comprehensive examinations that included a detailed questionnaire, anthropometric measurement, blood collection, and a standard 75-g oral glucose tolerance test (OGTT) or steamed-bread meal test. The self-administered questionnaire covered the history of diabetes, hypertension, hyperlipidaemia, acute/chronic nephritis, nephritic syndrome, kidney stones, cardiovascular diseases [CVD, including myocardial infarction (MI), stoke and coronary heart disease (CHD)], diabetes, alcohol intake, and smoking habits. Alcohol intake was classified as either consumption nearly/more than once a week currently or not; smoking habit was classified as smoking more than once a day or not.

Body mass indices (BMI) were calculated as the weight in kilograms divided by the height in metres squared (kg/m^2^). Repeated blood pressure (BP) was measured three times consecutively by the same observer in 5-min intervals. The average of three blood pressure measurements was used for analysis. Blood samples were collected by venipuncture, and all participants were told to fast for at least 10 h before the test. Participants with or without a history of diabetes underwent a 100 g steamed-bread meal test or 75 g oral glucose tolerance test, respectively. Fasting blood glucose (FBG), 2 h post-load blood glucose (PBG), serum triglycerides (TG), total cholesterol (TC), high-density lipoprotein cholesterol (HDL-C), low-density lipoprotein cholesterol (LDL-C), Haemoglobin A1c (HbA1c), alanine transferase (ALT), aspartate transferase (AST), gamma-glutamyl transferase (GGT), and Cr were measured in each centre. Non-high-density lipoprotein cholesterol (non-HDL-C) levels were calculated from the difference between serum TC and HDL-C.

The estimated glomerular filtration rate (eGFR) was expressed in mL/min per 1.73 m^2^ by the formula eGFR = 186 × [serum creatinine × 0.011] − 1.154 × [age] − 0.203 × [0.742 if female] × 1.233, where serum creatinine was expressed as μmol/L and 1.233 was the adjusting coefficient for the Chinese population. This formula is according to the abbreviated Modification of Diet in Renal Disease (MDRD), which was recalibrated for Chinese population [20].

### Definition of UACR group and blood lipids categories

The spot first morning urine samples were collected to measure the concentration of urine albumin and creatinine. UACR was calculated by dividing the urinary albumin concentrations by the urinary creatinine concentrations. The 8 centres adopted different UACR measurement methods, therefore, the normal value range and unit of the measurement were not unified. To avoid this problem, we divided UACR as classification variables for analysis. The UACR data were divided into the < 25% group, the 25–49% group, the 50–74% group, and the ≥ 75% group according to the quartile division of the centre where the subject visited. (UACR percentiles).

According to the guidelines for the prevention and treatment of dyslipidaemia in Chinese adults (revised in 2016), we classified blood lipids into the following categories. TC was grouped into three categories (appropriate: < 5.2 mmol/L, borderline high: 5.2–6.1 mmol/L, and high: ≥ 6.2 mmol/L), TG was grouped into three categories (appropriate: < 1.7 mmol/L, borderline high: 1.7–2.2 mmol/L, and high: ≥ 2.3 mmol/L), LDL-C was grouped into three categories (appropriate: < 3.4 mmol/L, borderline high: 3.4–4.0 mmol/L, and high: ≥ 4.1 mmol/L), non-HDL-C were grouped into three categories (appropriate: < 4.1 mmol/L, borderline high: 4.1–4.8 mmol/L, and high: ≥ 4.9 mmol/L) and HDL-C was grouped into two categories (normal: ≥ 1.0 mmol/L and low: < 1.0 mmol/L).

### Statistical analysis

The statistical analysis was performed using SPSS version 24.0 (IBM, Chicago, IL, USA). Continuous variables were presented as the means ± the standard deviations (SD) with the exception of skewed variables, which were presented as medians (interquartile ranges). Categorical variables were expressed as numbers (proportions). Differences in the continuous variables among the four subgroups of UACR were tested by one-way analysis of variance (ANOVA) followed by multiple comparison test using least significant difference (LSD). The χ2 test was used when the variables were categorical. Cardiovascular diseases (yes/no), smoking status and drinking status (non-current/current) were fitted as categorical variables.

The measured lipid parameters (i.e., TC, TG, HDL-C and LDL-C) and the calculated non-HDL-C were divided into groups according to Guidelines and the associations of these parameters among the quartiles of UACR were tested by ordered logistic regression analysis, performed in separate genders. Multivariate adjusted logistic regression analysis was also carried out to control potential confounders for determining the associations of lipid parameters with UACR in five models. Model 1 was adjusted for age and centres. Model 2 was further adjusted for ALT, AST, GGT and eGFR. Model 3 was further adjusted for current smoking, drinking status, and previously diagnosed CVD. Model 4 was further adjusted for BMI. Model 5 was further adjusted for HbA1c, SBP and DBP.

The relationship between TG and UACR was also explored within subgroups that were stratified by the level of eGFR (G1: eGFR ≥ 90 mL/min per 1.73 m^2^, G2: 60 ≤ eGFE < 90 mL/min per 1.73 m^2^, G3: eGFR < 60 mL/min per 1.73 m^2^), the level of BMI (Underweight: BMI < 18.5 kg/m^2^, Normal weight 18.5 ≤ BMI < 24 kg/m^2^, Overweight 24 ≤ BMI < 28 kg/m^2^, Obese BMI ≥ 28 kg/m^2^), the level of blood glucose (Normal: FBG < 5.6 and PBG < 7.8 mmol/L, Pre-diabetes: 5.6 ≤ FBG < 7.0 or 7.8 ≤ PBG < 11.1 mmol/L, Diabetes: FBG ≥ 7.0 or PBG ≥ 11.1 mmol/L), and level of blood pressure (Normal blood pressure: systolic blood pressure (SBP) < 120 and diastolic blood pressure (DBP) < 80 mmHg, High-normal blood pressure: 120 ≤ SBP < 140 and/or 80 ≤ DBP < 90mmHg, Hypertension: SBP ≥ 140 or DBP ≥ 90mmHg). In the stratified analyses, we separately examined feasible associated factors that could modify the relationship between albuminuria and lipid measures. Interaction analyses between TG and possible confounding factors were also conducted among the UACR groups. The odds ratios (OR) and the corresponding 95% confidence intervals (95% CI) were calculated. All statistical tests were two-sided, and P values < 0.05 were considered statistically significant.

## Results

### Clinical characteristics of the study population

A total of 34,569 subjects (11,390 men and 23,179 women) with a mean age (SD) of 57.9 (9.39) years were recruited. Table 1 shows the clinical and biochemical characteristics according to quartiles of UACR. There were 1127 (3.3%) participants with CHD, 387 (1.1%) participants with stroke, and 115 (0.3%) participants with MI. It can be seen that the incidence of cardiovascular disease increases with the increased UACR. The age of the participants increased as the UACR increased and the high quartile of UACR was characterized by significantly higher levels of blood glucose (0′,120′), BP, HbA1c, BMI, TC, LDL-C, non-HDL-C and TG. Furthermore, the eGFR decreased as the UACR increased.

**Table 1:** Characteristics of study population by UACR quartiles

Variable	Total	UACR quartile				P value
		Q 1 (n = 8645)	Q 2 (n = 8645)	Q 3 (n = 8645)	Q 4 (n = 8634)	
Age, years	57.89 ± 9.38	55.87 ± 8.67	57.07 ± 8.90	58.41 ± 9.32	60.20 ± 10.02	P < 0.001^a,b,c,d,e,f^
Male sex, no. (%)	11,390 (32.9)	3946 (45.6)	2779 (32.1)	2283 (26.4)	2382 (27.6)	P < 0.001
Current smoker, no. (%)	4308 (12.5)	1526 (17.7)	1054 (12.2)	827 (9.6)	901 (10.4)	P < 0.001
Current drinker, no. (%)	2394 (6.9)	803 (9.3)	563 (6.5)	531 (6.1)	497 (5.8)	P < 0.001
Previous MI, no. (%)	115 (0.3)	25 (0.3)	22 (0.3)	25 (0.3)	43 (0.5)	P = 0.021
Previous stroke, no. (%)	387 (1.1)	69 (0.8)	72 (0.8)	111 (1.3)	135 (1.6)	P < 0.001
Previous CHD, no. (%)	1127 (3.3)	190 (2.2)	261 (3.0)	269 (3.1)	407 (4.7)	P < 0.001
TG, mmol/L	1.32 (0.94, 1.32)	1.26 (0.90, 1.80)	1.30 (0.93, 1.88)	1.32 (0.95, 1.91)	1.43 (1.01, 2.09)	P < 0.001^b,c,e,f^
TC, mmol/L	4.99 ± 1.19	4.87 ± 1.17	4.98 ± 1.17	5.04 ± 1.19	5.07 ± 1.21	P < 0.001^a,b,c,d,e^
LDL-C, mmol/L	2.94 ± 0.92	2.87 ± 0.90	2.94 ± 0.91	2.97 ± 0.92	2.97 ± 0.94	P < 0.001^a,b,c,e^
HDL-C, mmol/L	1.30 ± 0.35	1.28 ± 0.35	1.31 ± 0.35	1.32 ± 0.35	1.28 ± 0.34	P < 0.001^a,b,e,f^
Non-HDL-C, mmol/L	3.69 ± 1.05	3.59 ± 1.03	3.68 ± 1.03	3.72 ± 1.05	3.78 ± 1.08	P < 0.001^a,b,c,d,e,f^
ALT, U/L	18.04 ± 13.91	17.98 ± 12.77	17.91 ± 13.49	17.71 ± 12.63	18.55 ± 16.36	P < 0.001^c,e,f^
AST, U/L	21.71 ± 12.44	21.24 ± 11.84	21.56 ± 11.04	21.58 ± 10.52	22.47 ± 15.66	P < 0.001^c,e,f^
GGT, U/L	28.73 ± 36.23	27.94 ± 34.17	27.49 ± 27.31	28.35 ± 37.02	31.12 ± 44.28	P < 0.001^c,e,f^
FBG, mmol/L	5.96 ± 1.70	5.71 ± 1.24	5.80 ± 1.40	5.95 ± 1.65	6.39 ± 2.24	P < 0.001^a,b,c,d,e,f^
PBG, mmol/L	8.45 ± 3.92	7.73 ± 3.15	8.06 ± 3.44	8.49 ± 3.93	9.52 ± 4.73	P < 0.001^a,b,c,d,e,f^
HbA1c, %	6.08 ± 1.04	5.87 ± 0.73	5.97 ± 0.86	6.09 ± 1.04	6.37 ± 1.37	P < 0.001^a,b,c,d,e,f^
SBP, mmHg	131.43 ± 20.30	126.43 ± 17.69	129.39 ± 19.17	131.89 ± 20.22	138.01 ± 22.07	P < 0.001^a,b,c,d,e,f^
DBP, mmHg	77.47 ± 10.82	76.25 ± 10.16	76.94 ± 10.42	77.24 ± 10.75	79.44 ± 11.64	P < 0.001^a,b,c,e,f^
BMI, kg/m^2^	24.58 ± 3.66	24.36 ± 3.44	24.41 ± 3.62	24.45 ± 3.57	25.09 ± 3.96	P < 0.001^c,e,f^
Cr, mmol/L	67.87 ± 16.75	69.16 ± 14.02	67.06 ± 16.01	66.33 ± 16.01	68.93 ± 20.17	P < 0.001^a,b,d,e,f^
eGFR, ml/min per 1.73 m^2^	95.39 ± 21.23	97.09 ± 21.10	96.13 ± 20.23	95.55 ± 20.74	92.80 ± 22.54	P < 0.001^a,b,c,e,f^

Data were mean ± SD or median (IQR) for skewed variables or numbers (proportions) for categorical variables

UACR: urinary albumin to creatinine ratio; MI: myocardial infarction; CHD: coronary heart disease; TG: triglycerides; TC: total cholesterol; LDL-C: low-density lipoprotein cholesterol; HDL-C: high-density lipoprotein cholesterol; Non-HDL-C: non-high-density lipoprotein cholesterol; ALT: alanine aminotransferase; AST: aspartate aminotransferase; GGT: gamma-glutamyl transferase; FBG: fasting blood glucose; PBG: postprandial blood glucose; HbA1c: haemoglobin A1c; SBP: systolic blood pressure; DBP: diastolic blood pressure; BMI: body mass index; Cr: serum creatinine; eGFR: estimated glomerular filtration rate

a, b, c, d, e, f: p < 0.05 for Q2 vs Q1, Q3 vs Q1, Q4 vs Q1, Q3 vs Q2, Q4 vs Q2, and Q4 vs Q3, respectively

### Associations of lipid parameters with quartiles of UACR

Tables 2, 3 and 4 shows OR and 95% CI of the UACR quartiles with the categories of TG, TC, LDL-C, HDL-C, and non-HDL-C after adjusting for multiple confounders, separated into men and women. As seen in these tables, compared with other lipid parameters, only TG showed a significant adjusted odds ratio for all subjects and both sexes in model 1–4. This relationship was attenuated after adjusting for HbA1c and BP, but TG ≥ 2.3 mmol/L was still significantly associated with UACR in both men and women whereas TC, LDL-C, HDL-C, and non-HDL-C were not (OR: 1.131, 95% CI 1.065–1.203, P < 0.001 in total subjects; OR: 1.134, 95% CI 1.022–1.258, P = 0.017 in men; OR: 1.129, 95% CI 1.046–1.219, P = 0.002 in women). Although TC ≥ 6.2 mmol/L was significantly associated with UACR in the fully-adjusted model, the association was only seen in women, and it was not as remarkable as the association with TG (Table 3). No significant and independent association between non-HDL-C or LDL-C and UACR.

**Table 2:** Associations between lipid parameters and UACR in the total subjects

Variable	Model 1		Model 2		Model 3		Model 4		Model 5	
	OR (95% CI)	P value	OR (95% CI)	P value	OR (95% CI)	P value	OR (95% CI)	P value	OR (95% CI)	P value
TG, mmol/L										
< 1.7	Reference		Reference		Reference		Reference		Reference	
1.7–2.2	*1.125 (1.066, 1.187)*	*< 0.001*	*1.118 (1.058, 1.182)*	*< 0.001*	*1.115 (1.055, 1.179)*	*< 0.001*	*1.088 (1.029, 1.151)*	*0.003*	1.022 (0.966, 1.081)	0.451
≥ 2.3	*1.301 (1.229, 1.377)*	*< 0.001*	*1.274 (1.200, 1.353)*	*< 0.001*	*1.285 (1.210, 1.364)*	*< 0.001*	*1.252 (1.179, 1.330)*	*< 0.001*	*1.131 (1.065, 1.203)*	*< 0.001*
TC, mmol/L										
< 5.2	Reference		Reference		Reference		Reference		Reference	
5.2–6.1	1.076 (1.009, 1.147)	0.026	1.056 (0.989, 1.128)	0.100	1.036 (0.970, 1.106)	0.289	1.047 (0.980, 1.118)	0.170	1.038 (0.971, 1.108)	0.274
≥ 6.2	1.018 (0.909, 1.140)	0.754	1.000 (0.891, 1.123)	0.996	0.950 (0.846, 1.067)	0.385	0.978 (0.871, 1.098)	0.706	0.966 (0.860, 1.086)	0.565
LDL-C, mmol/L										
< 3.4	Reference		Reference		Reference		Reference		Reference	
3.4–4.0	1.215 (1.001, 1.475)	0.049	1.230 (1.011, 1.498)	0.039	1.204 (0.989, 1.466)	0.065	1.213 (0.996, 1.477)	0.055	1.139 (0.934, 1.389)	0.199
≥ 4.1	1.249 (0.816, 1.912)	0.306	1.267 (0.828, 1.940)	0.276	1.238 (0.808, 1.897)	0.327	1.256 (0.820, 1.925)	0.295	1.215 (0.789, 1.870)	0.376
HDL-C, mmol/L										
≥1.0	Reference		Reference		Reference		Reference		Reference	
< 1.0	0.958 (0.909, 1.009)	0.107	0.963 (0.909, 1.021)	0.210	0.998 (0.941, 1.058)	0.942	0.975 (0.919, 1.034)	0.403	0.952 (0.897, 1.010)	0.106
Non-HDL-C, mmol/L										
< 4.1	Reference		Reference		Reference		Reference		Reference	
4.1–4.8	1.005 (0.936, 1.080)	0.882	1.013 (0.942, 1.089)	0.735	1.029 (0.957, 1.107)	0.435	1.011 (0.940, 1.087)	0.769	0.987 (0.917, 1.062)	0.731
≥ 4.9	1.098 (0.975, 1.237)	0.125	1.092 (0.967, 1.233)	0.155	1.125 (0.996, 1.271)	0.058	1.092 (0.966, 1.233)	0.158	1.035 (0.916, 1.170)	0.580

Model 1: adjusted for age, centres

Model 2: additionally adjusted for ALT, AST, GGT, eGFR

Model 3: additionally adjusted for smoking, drinking, MI, stroke, CHD

Model 4: additionally adjusted for BMI

Model 5: additionally adjusted for HbA1c, SBP, DBP

Italic values indicate significance of P value (P < 0.05)

UACR: urinary albumin to creatinine ratio; TG: triglycerides; TC: total cholesterol; LDL-C: low-density lipoprotein cholesterol; HDL-C: high-density lipoprotein cholesterol; Non-HDL-C: non-high-density lipoprotein cholesterol

**Table 3:** Associations between lipid parameters and UACR in men

Variable	Model 1		Model 2		Model 3		Model 4		Model 5	
	OR (95% CI)	P value	OR (95% CI)	P value	OR (95% CI)	P value	OR (95% CI)	P value	OR (95% CI)	P value
TG, mmol/L										
< 1.7	Reference		Reference		Reference		Reference		Reference	
1.7–2.2	*1.226 (1.116, 1.346)*	*< 0.001*	*1.178 (1.069, 1.298)*	*0.001*	*1.179 (1.070, 1.299)*	*0.001*	*1.142 (1.036, 1.259)*	*0.008*	1.075 (0.974, 1.186)	0.151
≥ 2.3	*1.408 (1.279, 1.549)*	*< 0.001*	*1.297 (1.172, 1.436)*	*< 0.001*	*1.292 (1.167, 1.430)*	*< 0.001*	*1.250 (1.129, 1.385)*	*< 0.001*	*1.134 (1.022, 1.258)*	*0.017*
TC, mmol/L										
< 5.2	Reference		Reference		Reference		Reference		Reference	
5.2–6.1	1.046 (0.920, 1.189)	0.492	1.018 (0.893, 1.160)	0.793	1.030 (0.903, 1.174)	0.660	1.041 (0.913, 1.186)	0.553	1.007 (0.882, 1.150)	0.916
≥ 6.2	0.943 (0.748, 1.188)	0.616	0.899 (0.711, 1.137)	0.376	0.910 (0.719, 1.151)	0.430	0.939 (0.742, 1.188)	0.599	0.872 (0.687, 1.107)	0.260
LDL-C, mmol/L										
< 3.4	Reference		Reference		Reference		Reference		Reference	
3.4–4.0	0.964 (0.617, 1.506)	0.872	0.939 (0.597, 1.476)	0.784	0.914 (0.580, 1.438)	0.697	0.941 (0.598, 1.482)	0.794	1.004 (0.635, 1.586)	0.987
≥ 4.1	1.309 (0.505, 3.393)	0.579	1.376 (0.530, 3.569)	0.512	1.437 (0.554, 3.728)	0.457	1.486 (0.573, 3.855)	0.416	1.623 (0.615, 4.284)	0.328
HDL-C mmol/L										
≥1.0	Reference		Reference		Reference		Reference		Reference	
< 1.0	*1.092 (1.008, 1.183)*	*0.030*	*1.109 (1.018, 1.208)*	*0.017*	*1.099 (1.008, 1.197)*	*0.031*	*1.060 (0.972, 1.155)*	*0.188*	1.065 (0.976, 1.162)	0.157
Non-HDL-C, mmol/L										
< 4.1	Reference		Reference		Reference		Reference		Reference	
4.1–4.8	0.966 (0.843, 1.108)	0.621	0.988 (0.859, 1.136)	0.863	0.987 (0.858, 1.134)	0.850	0.962 (0.837, 1.107)	0.592	0.954 (0.828, 1.099)	0.516
≥ 4.9	1.189 (0.943, 1.500)	0.143	1.184 (0.935, 1.498)	0.160	1.175 (0.928, 1.487)	0.181	1.128 (0.890, 1.428)	0.319	1.133 (0.893, 1.438)	0.304

Model 1: adjusted for age, centres

Model 2: additionally adjusted for ALT, AST, GGT, eGFR

Model 3: additionally adjusted for smoking, drinking, MI, stroke, CHD

Model 4: additionally adjusted for BMI

Model 5: additionally adjusted for HbA1c, SBP, DBP

Italic values indicate significance of P value (P < 0.05)

UACR: urinary albumin to creatinine ratio; TG: triglycerides; TC: total cholesterol; LDL-C: low-density lipoprotein cholesterol; HDL-C: high-density lipoprotein cholesterol; Non-HDL-C: non-high-density lipoprotein cholesterol

**Table 4:** Associations between lipid parameters and UACR in women

Variable	Model 1		Model 2		Model 3		Model 4		Model 5	
	OR (95% CI)	P value	OR (95% CI)	P value	OR (95% CI)	P value	OR (95% CI)	P value	OR (95% CI)	P value
TG, mmol/L										
< 1.7	Reference		Reference		Reference		Reference		Reference	
1.7–2.2	*1.098 (1.028, 1.173)*	*0.006*	*1.097 (1.025, 1.174)*	*0.008*	*1.093 (1.021, 1.170)*	*0.011*	*1.070 (0.999, 1.146)*	*0.053*	1.000 (0.933, 1.072)	0.994
≥ 2.3	*1.313 (1.222, 1.410)*	*< 0.001*	*1.296 (1.202, 1.397)*	*< 0.001*	*1.290 (1.197, 1.391)*	*< 0.001*	*1.263 (1.171, 1.362)*	*< 0.001*	*1.129 (1.046, 1.219)*	*0.002*
TC, mmol/L										
< 5.2	Reference		Reference		Reference		Reference		Reference	
5.2–6.1	0.954 (0.885, 1.028)	0.218	0.941 (0.872, 1.016)	0.123	0.944 (0.874, 1.019)	0.138	0.953 (0.883, 1.029)	0.222	0.941 (0.871, 1.017)	0.123
≥ 6.2	*0.806 (0.707, 0.920)*	*0.001*	*0.797 (0.696, 0.912)*	*0.001*	*0.798 (0.698, 0.913)*	*0.001*	*0.820 (0.716, 0.938)*	*0.004*	*0.812 (0.709, 0.930)*	*0.003*
LDL-C, mmol/L										
< 3.4	Reference		Reference		Reference		Reference		Reference	
3.4–4.0	*1.270 (1.023, 1.578)*	*0.031*	*1.300 (1.043, 1.620)*	*0.019*	*1.298 (1.042, 1.618)*	*0.02*	*1.303 (1.046, 1.624)*	*0.018*	1.206 (0.966, 1.506)	0.097
≥ 4.1	1.239 (0.768, 1.998)	0.379	1.301 (0.806, 2.100)	0.281	1.293 (0.801, 2.087)	0.294	1.308 (0.810, 2.112)	0.272	1.255 (.0.774, 2.036)	0.357
HDL-C, mmol/L										
≥ 1.0	Reference		Reference		Reference		Reference		Reference	
< 1.0	1.064 (0.990, 1.144)	0.09	1.044 (0.961, 1.135)	0.307	1.041 (0.958, 1.131)	0.347	1.023 (0.941, 1.112)	0.594	0.987 (0.908, 1.074)	0.765
Non-HDL-C, mmol/L										
< 4.1	Reference		Reference		Reference		Reference		Reference	
4.1–4.8	1.099 (1.010, 1.196)	0.029	1.103 (1.012, 1.203)	0.026	1.103 (1.012, 1.203)	0.026	1.086 (0.996, 1.184)	0.061	1.061 (0.973, 1.158)	0.18
≥ 4.9	*1.215 (1.055, 1.399)*	*0.007*	*1.200 (1.039, 1.385)*	*0.013*	*1.201 (1.040, 1.387)*	*0.013*	*1.171 (1.013, 1.352)*	*0.032*	1.103 (0.954, 1.275)	0.184

Model 1: adjusted for age, centres

Model 2: additionally adjusted for ALT, AST, GGT, eGFR

Model 3: additionally adjusted for smoking, drinking, MI, stroke, CHD

Model 4: additionally adjusted for BMI

Model 5: additionally adjusted for HbA1c, SBP, DBP

Italic values indicate significance of P value (P < 0.05)

UACR: urinary albumin to creatinine ratio; TG: triglycerides; TC: total cholesterol; LDL-C: low-density lipoprotein cholesterol; HDL-C: high-density lipoprotein cholesterol; Non-HDL-C: non-high-density lipoprotein cholesterol

### Associations between lipid parameters and UACR in people with LDL-C < 2.6 mmol/L or HDL-C > 1.0 mmol/L

According to the guidelines for the prevention and treatment of dyslipidaemia in Chinese adults (revised in 2016), we divided the population into two groups, LDL-C < 2.6 mmol/L (ideal value) and HDL-C > 1.0 mmol/L (low risk value). As shown in Table 5, high TG levels were still significantly associated with proteinuria excretion even if LDL-C was well controlled below 1.8 mmo/L or HDL-C was at a low risk level. No significant association was found in other lipid parameters.

**Table 5:** Associations between lipid parameters and UACR in people with LDL-C <  2.6 mmol/L or HDL-C > 1.0 mmol/L

Variable	LDL < 2.6 mmol/L		HDL > 1.0 mmol/L	
	OR (95% CI)	P value	OR (95% CI)	P value
TG, mmol/L				
< 1.7	Reference		Reference	
1.7–2.2	1.086 (0.977, 1.207)	0.126	1.031 (0.969, 1.097)	0.338
≥ 2.3	*1.144 (1.030, 1.269)*	*0.012*	*1.148 (1.069, 1.232)*	*< 0.001*
TC, mmol/L				
< 5.2	Reference		Reference	
5.2–6.1	1.076 (0.856, 1.353)	0.528	1.040 (0.971, 1.113)	0.259
≥ 6.2	0.673 (0.360, 1.256)	0.213	0.967 (0.857, 1.092)	0.589
LDL-C, mmol/L				
< 3.4	–		Reference	
3.4–4.0	–	–	1.140 (0.934, 1.393)	0.198
≥ 4.1	–	–	1.250 (0.807, 1.935)	0.318
HDL-C, mmol/L				
≥ 1.0	Reference		–	
< 1.0	0.985 (0.908, 1.068)	0.708	–	–
Non-HDL-C, mmol/L				
< 4.1	Reference		Reference	
4.1–4.8	0.997 (0.753, 1.319)	0.982	0.998 (0.923, 1.079)	0.958
≥ 4.9	1.279 (0.756, 2.164)	0.359	1.030 (0.904, 1.173)	0.654

Adjusted for age, sex, centres, ALT, AST, GGT, eGFR, MI, stroke, CHD, smoking, drinking, BMI, SBP, DBP, HbA1c

Italic values indicate significance of P value (P < 0.05)

UACR: urinary albumin to creatinine ratio; TG: triglycerides; TC: total cholesterol; LDL-C: low-density lipoprotein cholesterol; HDL-C: high-density lipoprotein cholesterol; Non-HDL-C: non-high-density lipoprotein cholesterol

### Associations of TG with quartiles of UACR and stratified analyses for different levels of eGFR, BMI, blood glucose and blood pressure

To verify the stability of such results, we conducted stratified analyses in the various subgroups as shown in Table 6. These results indicate that compared with subjects with TG levels < 1.7 mmol/L, subjects with TG levels ≥ 2.3 mmol/L have the most significant association with UACR, especially in the pre-diabetes population (5.6 ≤ FBG < 7.0 or 7.8 ≤ PBG < 11.1 mmol/L). However, no significant relationship was found in either the normal population (FBG < 5.6 and PBG < 7.8 mmol/L) or the diabetic population (FBG ≥ 7.0 or PBG ≥ 11.1 mmol/L). Similar results were seen in people with borderline high blood pressure (120 ≤ SBP < 140 and/or 80 ≤ DBP < 90 mmHg) and those who were overweight (24 ≤ BMI < 28 kg/m^2^). We found a significant interaction between TG and blood glucose. Therefore, we further divided the pre-diabetic population into the impaired fasting glucose group (IFG: 5.6 ≤ FBG < 7.0 mmol/L and PBG < 7.8 mmol/L) and the impaired glucose tolerance group (IGT: FBG < 7.0 mmol/L and 7.8 ≤ PBG < 11.1 mmol/L) to observe the effect of fasting and postprandial blood glucose on this interaction. As shown in Additional file 1: Table S2, there was a significant relationship between TG and UACR in IFG group.

**Table 6:** Associations between TG and UACR in people with different levels of eGFR, BMI, blood glucose and blood pressure

Variable	TG < 1.7 mmol/L	1.7 ≤ TG < 2.3 mmol/L		TG ≥ 2.3 mmol/L		P-values for interaction
	Reference	OR (95% CI)	P value	OR (95% CI)	P value	
eGFR, ml/min per 1.73 m^2 a^						0.504
eGFR ≥ 90	1	0.978 (0.902, 1.061)	0.589	*1.119 (1.008, 1.242)*	*0.035*	
60 ≤ eGFE < 90	1	1.014 (0.929, 1.108)	0.752	0.987 (0.881, 1.106)	0.824	
eGFR < 60	1	1.401 (0.912, 2.153)	0.124	1.609 (0.971, 2.667)	0.065	
BMI, kg/m^2^ ^b^						0.081
BMI < 18.5	1	1.472 (0.854, 2.539)	0.164	2.145 (0.866, 5.315)	0.099	
18.5 ≤ BMI < 24	1	1.016 (0.921, 1.120)	0.753	1.077 (0.944, 1.227)	0.270	
24 ≤ BMI < 28	1	1.000 (0.914, 1.093)	0.992	*1.153 (1.030, 1.291)*	*0.014*	
BMI ≥ 28	1	1.013 (0.881, 1.163)	0.861	0.933 (0.785, 1.107)	0.425	
Blood glucose, mmol/L^c^						*0.001*
FBG < 5.6 and PBG < 7.8	1	0.976 (0.881, 1.082)	0.648	1.089 (0.945, 1.255)	0.238	
5.6 ≤ FBG < 7.0 or 7.8 ≤ PBG < 11.1	1	1.001 (0.915, 1.096)	0.981	*1.139 (1.016, 1.277)*	*0.026*	
FBG ≥ 7.0 or PBG ≥ 11.1	1	1.095 (0.969, 1.237)	0.146	1.053 (0.909, 1.221)	0.489	
BP, mmHg^d^						0.068
SBP < 120 and DBP < 80	1	1.100 (0.975, 1.242)	0.12	1.044 (0.889, 1.226)	0.602	
120 ≤ SBP < 140 and/or 80 ≤ DBP < 90	1	1.048 (0.954, 1.152)	0.328	*1.145 (1.015, 1.290)*	*0.027*	
SBP ≥ 140 or DBP ≥ 90	1	0.968 (0.880, 1.066)	0.51	1.102 (0.976, 1.244)	0.116	

^a^Adjusted for SBP, DBP, BMI, HbA1c + age, sex, centres, ALT, AST, GGT, HDL-C, TC, LDL-C, non-HDL-C, MI, stroke, CHD, smoking, drinking

^b^Adjusted for SBP, DBP, HbA1c + age, sex, centres, ALT, AST, GGT, eGFR, HDL-C, TC, LDL-C, non-HDL-C, MI, stroke, CHD, smoking, drinking

^c^Adjusted for SBP, DBP, BMI,  + age, sex, centres, ALT, AST, GGT, eGFR, HDL-C, TC, LDL-C, non-HDL-C, MI, stroke, CHD, smoking, drinking

^d^Adjusted for BMI, HbA1c + age, sex, centres, ALT, AST, GGT, eGFR, HDL-C, TC, LDL-C, non-HDL-C, MI, stroke, CHD, smoking, drinking

Italic values indicate significance of P value (P < 0.05)

To better discuss the association of TG with UACR in different renal functions, we divided eGFR into three groups in Table 6. There was no significant association between TG and UACR in G2 stage (60 ≤ eGFR < 90 mL/min per 1.73 m^2^). However, when eGFR was more than 90 mL/min per 1.73 m^2^, the increase of triglyceride was significantly associated with UACR, while when eGFR was less than 60 mL/min per 1.73 m^2^, this relationship was at the borderline significant level. We also analyzed the relationship between all lipid indices and eGFR, as shown in Additional file 1: Table S1.

## Discussion

### Main findings

The main findings of this study suggested that among the lipid parameters, only TG was significantly associated with UACR in both men and women, whereas TC, LDL-C, HDL-C, and non-HDL-C were not. This association was consistently shown in the multiple regression models after adjusting for a wide spectrum of biochemical and lifestyle risk factors. To the best of our knowledge, the present study is the first multicentre, large sample clinical survey about the relationship between lipid parameters and UACR in a Chinese general population. After controlling for HbA1c and BP levels, the correlation between TG and UACR was weakened, indicating that HbA1c and BP levels added to the risk of proteinuria in this study. Further stratification showed that people with borderline values of BMI, blood glucose and BP had higher risks of urinary albumin when TG ≥ 2.3 mmol/L. Therefore, such people should be vigilant about the detection, avoidance and treatment of traditional risk factors.

### Factors associated with TG and UACR

This study also analyzed the interaction between TG and possible confounding factors in the UACR group. We found that there was a significant interaction between TG and blood glucose, especially in the group with impaired fasting glucose (P = 0.016, Additional file 1: Table S2). This finding suggests that FBG is more closely associated with TG and UACR in pre-diabetic populations. The results are consistent with another study of a Mexican population demonstrated that higher triglyceride levels, greater waist circumference, and smoking are risk factors associated to diabetic kidney disease [21]. In addition, a Japanese study also demonstrated that not only fasting TG and FBG, but also the management of postprandial TG may have important significance in preventing the progression of type 2 diabetic nephropathy [22].

Decreased β-cell function and insulin resistance (IR) are considered central events in the development of T2DM. TG overload in islets impairs the function of β cells and interferes with glucose metabolism [23, 24]. Recently, a prospective study reported that changes in TG have a unidirectional relationship with peripheral IR, which provides evidence for the early prevention of IR by improving dyslipidaemia [25]. Furthermore, study of patients with abnormal glucose metabolism added that after appropriate treatment of high TG and high FBG level by n-3 fatty acids in patients with impaired glucose metabolism, the ability of insulin secretion was improved [26]. An individual can have pre-diabetes without knowledge or diagnosis of it for many years. Unfortunately, they may start to develop complications like diabetic nephropathy during this time even when asymptomatic. In reality, this is often the case in most chronic diseases until the symptoms worsen to a point that it affects the individual [27]. The present findings may provide evidence for early prevention of diabetic nephropathy by improving hypertriglyceridemia [28].

Interestingly, we noted that higher eGFR levels had a closer link with TG and UACR in our study. Glomerular hyperfiltration (GH) has been reported as a predictor of overt diabetic nephropathy [29]. An estimated 70% and 50% of patients with type 1 and 2 diabetes, respectively, develop GH early in their disease [30]. This may support the observations from Serena Low et al. [31] on the relationship between baseline hyperfiltration and rapid renal progression in T2DM among multi-ethnic Asians population. It is believed that GH is caused first by alteration in tubule glomerular feedback and the activation of vasoactive mediators which increase glomerular capillary pressure and lead to secondary increases in GFR (detected as GH) [32]. The exact mechanisms have not been elucidated completely but there is evidence suggesting that amelioration of GH by blocking the renin-angiotensin aldosterone system may confer renoprotection. Therefore, it would be important for the clinic to identify individuals with GH and to intervene at the right time.

Additionally, previous studies have shown that eGFR has a U-shaped relationship with all-cause mortality, indicating the importance of both high and low eGFR [9, 33]. However, the association between TG and UACR in low eGFR group was at the borderline significant level (P = 0.065) in present study. This difference may be explained, in part, by insufficient sample size of low eGFR group in our study. Further large sample or prospective studies are necessary to clarify the association of TG with UACR in different levels of eGFR.

### Lipid parameters, atherosclerosis and proteinuria

It was widely accepted that high serum levels of LDL-C play a crucial role in the initiation and progression of atherosclerosis. Moreover, in the ACC/AHA and ESC/EAS guidelines, LDL-C is recommended as the most important lipid risk factor and therapeutic target for cardiovascular disease [34, 35]. However, LDL-C was not found to be a good indicator for albuminuria in present research (Tables 2, 3 and 4). It should be noted that measurement of LDL-C concentration has neglected the impact of other highly atherogenic particles such as very low-density lipoprotein (VLDL), and intermediate-density lipoprotein (IDL). Lipoprotein (a) and lipoprotein (b), which are called remnant cholesterol, also contribute to the development of atherosclerosis [36]. Furthermore, Assmann et al. have proved that despite currently available optimal LDL-C lowering therapies, a worrisome number of clinical events still occur [37, 38]. In fact, even if LDL was well controlled below 1.8 mmo/L, hypertriglyceridemia was still significantly associated with proteinuria excretion in this study (OR: 1.144, 95% CI 1.030–1.269, P = 0.012, Table 5). And the contribution of other lipid subfractions is increasingly being recognized [39, 40].

Interestingly, we found that HDL-C < 1.0 mmol/L was significantly associated with increased UACR in men after adjustment for many confounders, but this association was abrogated after additional adjustment for HbA1c and BP. High levels of HDL-C were thought to be protective against the development of atherosclerosis, and a low HDL-C level was associated with increased risk of CHD [41]. Additionally, some researchers hold that elevated triglyceride concentrations are strongly associated with low concentrations of HDL-C [42]. However, similar results were not observed in the present study. In fact, we used logistic regression to adjust for HDL-C (both separately and simultaneously), LDL-C, non-HDL-C, TC, BMI, BP, HbA1c, eGFR and other traditional risk factors for arteriosclerosis to reduce the possibility that these factors would confound our results. However, TG remained independently significantly associated with albuminuria. Apart from this, large outcome trials using fibrate or cholesterol ester transfer protein (CETP) inhibitors were designed to increase HDL-C and reduce the incidence of cardiovascular events. However, the overall results were negative or only positive in the subgroup. Thus, low HDL-C was not causally linked to atherosclerotic events on a population level. In fact, low HDL-C may simply be a very good indicator for an increased concentration of triglyceride-rich lipoproteins (TGRL) [43].

The understanding from recent genetic studies and randomized trials that low HDL-C might not be a cause of atherosclerotic disease as originally thought has generated renewed interest in elevated triglycerides [44]. Meta-analyses have supported the findings that high concentrations of TG were associated with increased risk of atherosclerotic disease even after adjustment for HDL-C concentrations [45]. Two reports from Korea also pointed out that TG has a strong association with arteriosclerosis in Korean adults [46, 47]. In addition, genetic studies strongly support the theory that high concentrations of TGRL or remnant cholesterol are causal risk factors for cardiovascular disease [48, 49]. It has been reported that a doubling of genetically elevated non-fasting triglyceride concentrations due to APOA5 genetic variants was associated with a 1.9-fold increased risk of myocardial infarction [50]. Specifically, the lipoprotein lipase (LPL) pathway and its reciprocal regulators apoA-V and apoC-III have been found to have remarkable associations with both TG and CHD [51].

### The association and potential physiopathological mechanisms between TG and UACR

The role of dyslipidaemia in the development of albuminuria is still controversial, and the results of related studies have been inconsistent so far. One study of 275 Taiwanese cases reported that ApoB was the highest risk factor for albuminuria in both the diabetic men and the diabetic women [52]. However, the cases in this study involved early stage albuminuria, and the Cr levels were lower than 1.2 mg/dL. The exclusion of patients with elevated levels of Cr made the research results less reliable. Another prospective study from the Steno Diabetes Centre reported the baseline TC but not HDL was considered to be an independent risk factor for both micro-albuminuria and macro-albuminuria in type 2 diabetic patients. However, this study evaluated patients with more advanced renal disease [53].

Our findings are consistent with a Taiwanese study that recruited more participants (1026 males, 1323 females) and concluded that TG increased significantly throughout the 3 stages of albuminuria in Taiwanese Type 2 diabetic patients [54]. Additionally, an American study citing data from CACTI Study also showed that in adults with type 1 diabetes, fasting TG independently predicted higher odds of both coronary artery calcification (CACp) and incident albuminuria over 6 years, whereas LDL-C, HDL-C, non-HDL-C and TC did not. However, most of these studies were in people with diabetes. In our study, we confirmed the association between TG and UACR in the general population in 8 regions of China. Compared with the above studies, our study population was much larger, which allowed a careful control for the potential confounding effects using a stratified analysis and detected significant associations between TG and UACR in different levels of eGFR, BMI, blood glucose and BP.

A lipid profile includes measurement of the total amount of the two most important lipids in the plasma compartment–cholesterol and triglycerides. Lipoproteins include the smallest lipoproteins, HDL; medium-sized lipoproteins, LDL; and the largest lipoproteins, triglyceride-rich lipoproteins (remnants). For clinical reasons, the cholesterol content in these lipoprotein classes was reported as: HDL cholesterol, LDL cholesterol, and remnant cholesterol. We defined remnant cholesterol as the cholesterol content of all triglyceride-rich lipoproteins, i.e., chylomicron remnants, VLDL, and IDL.

Although the underlying pathophysiological mechanisms responsible for lipid-induced renal damage have yet to be uncovered, several studies suggest novel mechanisms by which TG may affect glomerular and tubular cell function. Plasma TG levels are known to correspond with the levels of TGRLs and their remnants [55]. The infiltration of TGRL into the glomerular endothelium and mesangial cells can trigger a cascade of events, including TGF-β pathway activation, monocyte chemoattractant production, adhesion molecule expression, and release of reactive oxygen species, that lead to early glomerular injury [56]. Once in the intima, LPL activity at the surface of remnants, either at the vascular endothelium or within the intima, leads to liberation of free fatty acids, monoacylglycerols, and other molecules, as well as foam cell formation, each of which could cause local injury and inflammation [57]. There is evidence that cellular damage by fatty acid accumulation in the kidney is particularly severe in podocytes, leading to apoptosis and resulting in glomerulosclerosis [58].

## Limitations

Our study benefited from a large aggregation of multiple community-based samples, and the distribution of different regions in China was generally representative. However, some limitations existed. First, due to the different UACR measurement methods that were adopted by the 8 centres, the measurement units of UACR were not unified. As a result, the UACR could not be expressed as a continuous variable in the statistical analyses. However, the relationship between TG and UACR persisted after controlling for traditional risk factors in ordinal regression analysis. Second, although we excluded subjects who used ACEI/ARB and lipid-lowering drugs, we did not investigate other medications which might affect this relationship. Therefore, we could not eliminate the possible effect of medications on the present findings. Third, we evaluated the urinary albumin excretion on a spot morning urine sample. We admitted that multiple samples would provide more stable results for albumin excretion. However, it was reported that the results of spot urine samples correlate well with those of 24 h or multiple urine samples. The use of spot samples for assessing urinary ACR is more convenient and therefore recommended as a reliable alternative to perform in large epidemiological specimen collection [59]. Finally, as a cross-sectional study, we can only establish associations not cause. Further prospective follow-up studies are needed to fully ascertain the mechanisms underlying the association between dyslipidaemia and albuminuria.

## Conclusion

In summary, we observed that high TG levels rather than total cholesterol, low-density lipoprotein cholesterol, high-density lipoprotein cholesterol, or non-high-density lipoprotein cholesterol levels are associated with UACR in the general population in China. People whose eGFR ≥ 90 mL/min per 1.73 m^2^ and BMI, blood glucose, or BP were borderline abnormal were more likely to have high risk of urinary albumin when TG ≥ 2.3 mmol/L. Given the clinical correlations with dyslipidaemia and proteinuria, it is important to take effective methods to improve the dyslipidaemia to decrease the risk of cardiovascular mortality and CKD progression. We believe that targeting lipid metabolism disorders in renal disease may increase the chance of successful drug discovery in the field of proteinuric kidney diseases.

## Additional file

**Additional file 1: Table S1.** Associations between lipid parameters and eGFR in the total subjects, men and women. **Table S2.** Associations between TG and UACR in pre-diabetic population.

## Abbreviations

UACR: urinary albumin to creatinine ratio
TG: triglycerides
HbA1c: haemoglobin A1C
BP: blood pressure
TC: total cholesterol
LDL-C: low-density lipoprotein cholesterol
HDL-C: high-density lipoprotein cholesterol
non-HDL-C: non-high-density lipoprotein cholesterol
eGFR: estimated glomerular filtration rate
CKD: chronic kidney disease
T2DM: type 2 diabetes mellitus
ACEI: angiotensin converting enzyme inhibitor
ARB: angiotensin II receptor blocker
CVD: cardiovascular diseases
MI: myocardial infarction
CHD: coronary heart disease
Cr: serum creatinine
FBG: fasting blood glucose
PBG: 2 h post-load blood glucose
ALT: alanine transferase
AST: aspartate transferase
GGT: gamma-glutamyl transferase
SBP: systolic blood pressure
DBP: diastolic blood pressure
ACC/AHA: American College of Cardiology and American Heart Association
ESC/EAS: European Society of Cardiology and European Atherosclerosis Society
IR: insulin resistance
IFG: impaired fasting glucose
IGT: impaired glucose tolerance
GH: glomerular hyperfiltration
TGRL: triglyceride-rich lipoproteins
