# Supplementary material for: Elevated triglycerides rather than other lipid parameters are associated with increased urinary albumin to creatinine ratio in the general population of China: a report from the REACTION study
Source: Cardiovasc Diabetol. 2019 May 4;18:57. doi: 10.1186/s12933-019-0863-8 (PMC6500581; doi:10.1186/s12933-019-0863-8)
Supplement: Supplementary file 1 — Additional file 1: Table S1. Associations between lipid parameters and eGFR in the total subjects, men and women. Table S2. Associations between TG and UACR in pre-diabetic population. [file 12933_2019_863_MOESM1_ESM.docx]

**Additional Table S1 Associations between lipid parameters and eGFR in the total subjects, men and women**

| **Variable** | **Total** | | **Men** | | **Women** | |
| --- | --- | --- | --- | --- | --- | --- |
|  | **OR (95% CI)** | **P value** | **OR (95% CI)** | **P value** | **OR (95% CI)** | **P value** |
| **TG, mmol/L** | | | | | | |
| <1.7 | Reference | | Reference | | Reference | |
| **1.7-2.2** | **1.355(1.268,1.447)** | **<0.001** | **1.349(1.200,1.516)** | **<0.001** | **1.329(1.225,1.441)** | **<0.001** |
| **≥2.3** | **1.723(1.604,1.851)** | **<0.001** | **1.518(1.342,1.718)** | **<0.001** | **1.783(1.630,1.950)** | **<0.001** |
| TC, mmol/L | | | | | | |
| <5.2 | Reference | | Reference | | Reference | |
| 5.2-6.1 | 1.374(1.271,1.486) | <0.001 | 1.158(0.988,1.358) | 0.071 | 1.348(1.230,1.477) | <0.001 |
| ≥6.2 | 1.439(1.256,1.649) | <0.001 | 1.120(0.847,1.482) | 0.426 | 1.365(1.164,1.600) | <0.001 |
| LDL-C, mmol/L | | | | | | |
| <3.4 | Reference | | Reference | | Reference | |
| 3.4-4.0 | 1.172(0.935,1.470) | 0.169 | 0.932(0.552,1.573) | 0.792 | 1.265(0.979,1.636) | 0.072 |
| ≥4.1 | 1.803(1.097,2.963) | 0.020 | 1.005(0.310,3.257) | 0.993 | 2.191(1.240,3.872) | 0.007 |
| HDL-C, mmol/L | | | | | | |
| ≥1.0 | Reference | | Reference | | Reference | |
| <1.0 | 0.560(0.520,0.602) | <0.001 | 0.740(0.665,0.822) | <0.001 | 0.457(0.412,0.507) | <0.001 |
| Non-HDL-C, mmol/L | | | | | | |
| <4.1 | Reference | | Reference | | Reference | |
| 4.1-4.8 | 1.103(1.012,1.202) | 0.025 | 1.112(0.940,1.317) | 0.216 | 1.145(1.034,1.268) | 0.009 |
| ≥4.9 | 1.126(0.976,1.299) | 0.104 | 1.315(0.994,1.741) | 0.055 | 1.119(0.945,1.326) | 0.193 |
| eGFR: estimated glomerular filtration rate; UACR: urinary albumin to creatinine ratio; TG: triglycerides; TC: total cholesterol; LDL-C: low-density lipoprotein cholesterol; HDL-C: high-density lipoprotein cholesterol; Non-HDL-C: non-high-density lipoprotein cholesterol; | | | | | | |
| Adjusted for age, centres, ALT, AST, GGT, UACR , MI, stroke, CHD, smoking, drinking, BMI, SBP, DBP, HbA1c | | | | | | |

**Additional Table S2 Associations between TG and UACR in pre-diabetic population**

| **Variable** | **TG<1.7 mmol/L** | **1.7≤TG<2.3 mmol/L** | | **TG≥2.3 mmol/L** | |
| --- | --- | --- | --- | --- | --- |
|  | Reference | OR (95% CI) | P value | OR (95% CI) | P value |
| IFG: 5.6≤FBG<7.0 and PBG<7.8 | 1 | 1.034(0.887,1.206) | 0.669 | **1.277(1.048,1.558)** | **0.016** |
| IGT: FBG<7.0 and 7.8≤PBG<11.1 | 1 | 0.981(0.876,1.097) | 0.732 | 1.057(0.917,1.217) | 0.446 |
| IFG: impaired fasting glucose; IGT: impaired glucose tolerance; FBG: fasting blood glucose; PBG: 2 h post-load blood glucose; TG: triglycerides.  Adjusted for SBP, DBP, BMI + age, sex, centres, ALT, AST, GGT, eGFR, TC, LDL-C, HDL-C, non-HDL-C, MI, stroke, CHD, smoking, drinking | | | | | |
